# Sex-specific risk prediction models for aneurysmal subarachnoid hemorrhage—A UK Biobank study

**DOI:** 10.1177/17474930251349928

**Published:** 2025-06-07

**Authors:** Ina Rissanen, Vita M Klieverik, Jos P Kanning, Mirjam I Geerlings, Ynte M Ruigrok

**Affiliations:** 1 Julius Centre for Health Sciences and Primary Care, University Medical Centre Utrecht, Utrecht, The Netherlands; 2 Department of Neurology and Neurosurgery, University Medical Centre Utrecht Brain Centre, University Medical Centre Utrecht, Utrecht, The Netherlands; 3 Amsterdam UMC, Department of General Practice, University of Amsterdam, Amsterdam, The Netherlands; 4 Amsterdam Public Health; Aging & Later life, and Personalized Medicine, Amsterdam, The Netherlands; 5 Amsterdam Neuroscience; Neurodegeneration, and Mood, Anxiety, Psychosis, Stress, and Sleep, Amsterdam, The Netherlands

**Keywords:** Prediction, subarachnoid hemorrhage, sex differences, risk calculator

## Abstract

**Background:**

We recently developed and validated the SMA^2^SH^2^ERS risk prediction model for aneurysmal subarachnoid hemorrhage (ASAH) in the general population (c-statistic 0.62; 95% confidence interval [CI] 0.60–0.64). Given that women have higher ASAH incidence than men, and that predictors for ASAH have different effect sizes between sexes, we developed sex-specific risk prediction models.

**Methods:**

Data from the prospective UK Biobank Study were used for model development. Participants with ASAH (per hospital-based ICD codes) before baseline or with missing predictor data were excluded. We developed multivariable Cox proportional hazards models for women and men separately to study the association between earlier recognized SMA^2^SH^2^ERS predictors and incident ASAH. Predictive performances were assessed with c-statistics and calibration plots and corrected for overfitting using bootstrapping.

**Results:**

A total of 246,771 women and 210,085 men were included with median follow-up of 12 years. ASAH incidence rate per 100 000 person years was 16.1 in women, and 10.7 in men. The women-specific model had a c-statistic of 0.63 (95% CI 0.60–0.65) and the mean predicted absolute 10-year ASAH risk was 0.15%. Independent predictors for women were higher age, family history of stroke, former and current smoking, alcohol consumption, and intermediate education. The men-specific model c-statistic was 0.57 (95% CI 0.53–0.60) and the mean 10-year risk 0.10%. Independent predictors for men were higher age, hypertension, and smoking status.

**Conclusion:**

The sex-specific models did not perform better than the general SMA^2^SH^2^ERS model in women or in men. Further validation studies are needed before clinical use can be recommended.

## Graphical abstract

**Graphical abstract:**
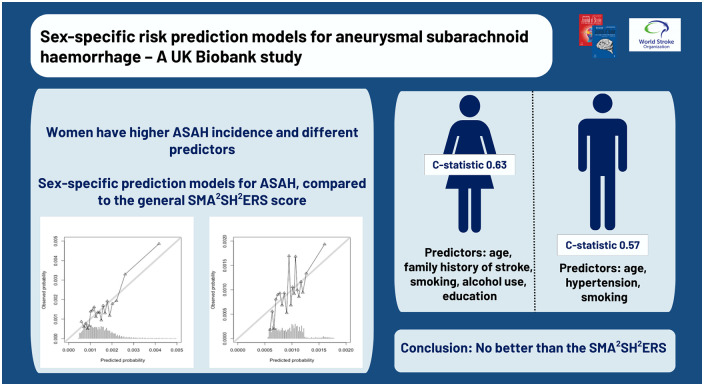

## Introduction

The incidence of aneurysmal subarachnoid hemorrhage (ASAH) is different between sexes, as two thirds of ASAH patients are women.^1^ Women have a higher risk of developing ASAH, both as patients previously diagnosed with an unruptured intracranial aneurysm (UIA),^2^ and when the presence of aneurysm is unknown.^3^ Sex differences in ASAH incidence are at least partly explained by the differences in risk factors, since common ASAH risk factors have a different impact on the disease development in women and men.^4^ For example, smoking and hypertension pose a greater excess risk for ASAH in women than in men.^5,6^ Moreover, previous studies have also suggested sex-specific risk factors for ASAH, such as use of oral contraceptives and menopause.^7^

We have recently published the SMA^2^SH^2^ERS risk prediction model that predicts ASAH in the general population and helps to identify persons with an up to 12 times increased risk of ASAH.^8^ The independent predictors in the prediction model were Sex (S), diabetes mellitus (M), Age and Alcohol consumption (A^2^), Smoking (S), Hypertension and Hypercholesterolemia (H^2^), Educational attainment (E), Regular physical activity (R) and family history of Stroke (S; SMA^2^SH^2^ERS), and interactions between these predictors. Predictors age, hypertension, and smoking had interactions with sex suggesting a different prediction for women and men.

The performance of a disease prediction model may improve by developing sex-specific models using the same predictors,^9^ particularly when sex differences exist in incidence or risk factors, as is the case in ASAH.^1,4^ In addition, sex-specific prediction models are necessary when predictors have different effect sizes, as observed in our SMA^2^SH^2^ERS risk prediction model,^8^ where age, hypertension, and smoking interact with sex, suggesting differential associations in women and men.

We developed ASAH prediction models in women and men separately and assessed whether these sex-specific models improve ASAH prediction by assessing model discrimination and calibration. The study hypothesis was that sex-specific prediction models would perform in each sex category better than a non-sex-specific prediction model.

## Methods

Similarly to the SMA^2^SH^2^ERS risk prediction model,^8^ we used data from the United Kingdom Biobank Prospective Cohort Study (UKBB) for the development of the sex-specific models. UKBB is a large general population-based study containing over 500 000 participants aged 37 to 73 years and recruited from 2006 to 2010. These participants attended 22 assessment centers across England, Scotland, and Wales, where they were interviewed and had physical measurements taken.^10^ All participants provided their written informed consent, and the study was approved by the North West Multicenter Research Ethics Committee and the National Health Service National Research Ethics Service (ref 11/NEW/0382). Permission to use the UKBB data was obtained under a UKB project 2532 UK Biobank Stroke Study (UKBiSS): Developing an in-depth understanding of the determinants of stroke and its subtypes.

### Outcome

Data from the UKBB were subsequently linked to National Health Service data, including inpatient hospital and primary care data to record diagnosis information. At the time of analysis, the follow-up data from participants were available up to 28 March 2021. The primary outcome was incident non-traumatic ASAH, ascertained using the International Classification of Disease version 9 (ICD-9) code 430 and version 10 (ICD-10) codes I600 to I609. The diagnoses were derived from hospital register data. Participants with a previous history of ASAH at baseline assessment (n = 792) were excluded from this study (Supplemental Figure 1).

### Candidate predictors

Candidate predictors were all the same predictors that were tested in our previous development study of the SMA^2^SH^2^ERS risk prediction model,^8^ i.e. predictors that are routinely available or can be easily ascertained by general practitioners during a standard consultation. These included age, family history of stroke, hypertension, smoking status, hypercholesterolemia, regular physical activity, hormone replacement therapy (HRT) for women, diabetes mellitus (DM), alcohol consumption, and educational attainment. The candidate predictors were assessed once at baseline and held static. Sex was used to stratify the population into women and men.

A family history of stroke was defined as at least one first-degree relative being affected with the disease. Hypertension was defined as systolic blood pressure ⩾ 140 mmHg or diastolic blood pressure ⩾ 90 mmHg and/oruse of antihypertensive medication. We categorized smoking status into (1) never smokers (reference), (2) former smokers, and (3) current smokers. Hypercholesterolemia was defined as use of cholesterol lowering medication. Regular physical activity was defined as vigorous physical activity ⩾ three times per week. We grouped HRT into (1) never users (reference) and (2) former and current users combined. DM was defined based on a past medical history of DM and/or use of antidiabetic medication. We categorized alcohol consumption into (1) no alcohol consumption, (2) alcohol consumption on special occasions (reference), and (3) daily or almost daily alcohol consumption. Educational attainment was used as a surrogate for socioeconomic status. We grouped educational attainment into (1) high, (2) intermediate (reference), and (3) low. High educational attainment was defined as having a university or college degree, intermediate educational attainment as having either an Advanced Level qualification, Ordinary Level qualification, Certificate of Secondary Education (CSE), National Vocational Qualification (NVQ), Higher National Diploma (HND), Higher National Qualification (HNC), or other professional qualification, and low educational attainment as having no degree.

No predictor variables had missing values > 6% (Supplement Table 1). Since the proportion of missing data was small and its potential impact was expected to be minor, participants with missing data on any of the candidate predictors (7.5% of participants) were excluded from the analysis (Supplemental Figure 1).

### Statistical analyses

In baseline characteristics, continuous variables were expressed as means ± standard deviations (SD) and categorical variables as counts with corresponding percentages. Differences in baseline characteristics between women and men were tested using t-tests for continuous variables and Chi-square tests for categorical variables.

We developed multivariable Cox regression models for incident ASAH based on the candidate predictors separately for women and men, using time in study as time scale. Follow-up data were censored at time of incident ASAH, death, or last follow-up assessment on 28 March 2021, whichever came first. The functional form (i.e. linearity) of the continuous candidate predictor age was assessed using martingale residuals. Candidate predictors were entered into the models irrespective of their association with incident ASAH in univariable analyses. To assess whether candidate predictors contributed to the models, we performed backward selection based on Akaike Information Criterion (AIC). Due to low numbers of ASAH cases, especially among men, no predictor interactions were included for either women or men.

We assessed the proportional hazard assumption visually and numerically using scaled Schoenfeld residuals plots and tests. Prediction models derived from multivariable regression may be overfitted to the development cohort and thus overestimate effect sizes when applied to a different population. To correct for this, we applied shrinkage factors to the regression coefficients, determined by bootstrapping procedures. The estimated effect sizes of the independent predictors derived from the models were expressed as hazard ratio’s (HR) with corresponding 95% confidence intervals (CI).

Performances of the sex-specific models were assessed using discrimination and calibration. Discrimination indicates a model’s ability to correctly distinguish between persons with and without ASAH and we evaluated this ability using the concordance statistic (c-statistic). The c-statistic is the probability that, given two individuals: one who gets an ASAH and one who does not, the model will yield a higher risk for the first individual than for the second.^11^ C-statistics generally range from 0.5 (random concordance) to 1 (perfect concordance). We corrected the c-statistic for overoptimism using bootstrapping procedures. In addition to sex-specific models, we estimated the performance of the original SMA^2^SH^2^ERS risk prediction model^8^ separately in women and men. The difference between the c-statistic of each sex-specific model and the c-statistics of the SMA^2^SH^2^ERS risk prediction model in that sex category was calculated before overoptimism correction as ΔC = C_sex-specific model_ − C_SMA2SH2ERS_. 95% confidence intervals for ΔC were estimated from the bootstrapping procedures. Similarly, difference in the c-statistic of the SMA^2^SH^2^ERS risk prediction model was compared between women and men before overoptimism correction as ΔC = C_SMA2SH2ERS in women_ − C_SMA2SH2ERS in men_. Calibration is an indicator for the agreement between predicted and observed probability of incident ASAH and we assessed this visually with 5 year and 10 year calibration plots. All statistical analyses were performed using R statistical software, version 4.2.2 (R Foundation for Statistical Computing, Vienna, Austria).

## Results

### Characteristics of sample

The baseline characteristics of women and men in the UKBB are presented in Table 1. A total of 246 771 women and 210 085 men were included in the analysis. During the follow-up of 2 941 645 person years 474 (0.2%) women developed ASAH; and during 2 466 265 person years 264 (0.1%) men developed ASAH. The incidence rate was 16.1 per 100 000 person years in women, and 10.7 per 100 000 person years in men. The median follow-up was similar in women and men (12.1 years (range 0.0 to 14.3) vs 12.0 years (range 0.0 to 14.3), respectively).

**Table 1.** Baseline characteristics of participants.

Characteristic	All (n = 456,856)		Women (n = 246,771)		Men (n = 210,085)		
	n	%	n	%	n	%	p-value
**Age (years)**							
< 50 years	109,062	23.9	59,676	24.2	49,386	23.5	< 0.001
⩾ 50 years	347,794	76.1	187,095	75.8	160,699	76.5	
Mean ± SD	56.4 ± 8.1	56.2 ± 8.0	56.6 ± 8.2	< 0.001			
**Positive family history for stroke**	119,690	26.2	67,019	27.2	52,671	25.1	< 0.001
**Hypertension**	227,823	49.9	107,033	43.4	120,790	57.5	< 0.001
**Smoking status**							
Never smoking	250,870	54.9	147,133	59.6	103,737	49.4	< 0.001
Former smoking	159,174	34.8	78,286	31.7	80,888	38.5	
Current smoking	46,812	10.2	21,352	8.7	25,460	5.6	
**Hypercholesterolemia**	77,510	17.0	29,980	12.1	47,530	22.6	< 0.001
**Having regular physical exercise**	148,892	32.6	72,034	29.2	76,868	36.6	< 0.001
**Hormone replacement therapy**	93,223	20.4	93,223	37.8	n.a.	n.a.	n.a.
**Diabetes mellitus**	22,903	5.0	8,827	3.6	14,076	6.7	< 0.001
**Alcohol consumption**							
Never	34,201	7.5	21,769	8.8	12,432	5.9	< 0.001
On special occasions	327,445	71.7	184,098	74.6	142,347	68.2	
Daily or almost daily	95,210	20.8	40,904	16.6	54,306	25.8	
**Educational attainment**							
Low	71,427	15.6	37,767	15.3	33,660	16.0	< 0.001
Intermediate	230,438	50.4	128,033	51.9	102,405	48.7	
High	154,991	33.9	80,971	32.8	74,020	35.2	

Data are n (%), unless otherwise indicated. SD = standard deviation, n.a. = not applicable. P-value from Chi Square test or t-test for difference between women and men.

### Development and performance of sex-specific models in women and men

Both in women and men, the martingale residuals showed that age could be analyzed as a linear predictor (not shown). We inspected the scaled Schoenfeld residuals plots and tests for each independent predictor and detected no deviations from the proportional hazard assumption (Supplemental Figures 2 and 3).

In women, independent predictors for ASAH were age, family history of stroke, former and current smoking, alcohol consumption, and educational attainment (Table 2). Hypertension, hypercholesterolemia, regular physical exercise, hormone replacement therapy, and diabetes mellitus were excluded from the model because of their limited predictive value for incident ASAH. The regression equation of the model is: ASAH hazard ~ 0.04 * age + 0.15 family history – 0.03 * former smoking + 0.94 * current smoking + 0.34 * never alcohol use + 0.09 * daily alcohol use – 0.07 low education – 0.27 * high education.

**Table 2.** Univariable and multivariable Cox proportional hazards regression analysis of predictors of incident ASAH in women.

Predictor	Univariable	Multivariable^a^
	HR (95% CI)	HR (95% CI)
Age per year	1.04 (1.03–1.06)	1.04 (1.03–1.05)
Family history of stroke	1.29 (1.07–1.57)	1.17 (0.98–1.39)
Smoking status		
Never smoking	Reference	
Former smoking	1.02 (0.83–1.26)	0.97 (0.80–1.18)
Current smoking	2.61 (2.05–3.31)	2.56 (2.05–3.21)
Alcohol consumption		
Never	1.58 (1.20–2.08)	1.41 (1.09–1.82)
On special occasions	Reference	
Daily or almost daily	1.18 (0.93–1.50)	1.10 (0.88–1.37)
Educational attainment		
Low	1.22 (0.97–1.54)	0.93 (0.75–1.17)
Intermediate	Reference	
High	0.68 (0.54–0.84)	0.76 (0.62–0.93)

a The initial multivariable regression coefficients were corrected for overfitting with a shrinkage factor of 0.9229. HR = hazard ratio, CI = confidence interval.

Following shrinkage of the regression coefficients with shrinkage factor of 0.92, the c-statistic of the women-specific model was 0.63 (95% CI 0.60–0.65). The 5 year and 10 year calibration plots showed good correspondence between predicted and observed risk (Supplemental Figure 4). The mean predicted 5 year absolute risk of ASAH was 0.07% and ranged from the minimum of 0.02% to the maximum of 0.40%. The mean predicted 10 year cumulative absolute risk was 0.15% and ranged from 0.05% to 0.91%.

In men, independent predictors were age, hypertension, and smoking status (Table 3). Family history for stroke, hypercholesterolemia, regular physical exercise, diabetes mellitus, alcohol consumption, and educational attainment were excluded from the model because of their limited predictive value for incident ASAH. The regression equation of the model is: ASAH hazard ~ 0.01 * age + 0.32 * hypertension + 0.11 * former smoking + 0.47 * current smoking.

**Table 3.** Univariable and multivariable Cox proportional hazards regression analysis of predictors of incident ASAH in men.

Predictor	Univariable	Multivariable^a^
	HR (95% CI)	HR (95% CI)
Age per year	1.02 (1.01–1.04)	1.01 (1.00–1.02)
Hypertension	1.61 (1.24–2.08)	1.38 (1.12–1.69)
Smoking status		
Never smoking	Reference	
Former smoking	1.25 (0.96–1.64)	1.11 (0.90–1.37)
Current smoking	1.80 (1.28–2.53)	1.60 (1.23–2.08)

a The initial multivariable regression coefficients were corrected for overfitting with a shrinkage factor of 0.7690. HR = hazard ratio, CI = confidence interval.

Following shrinkage of the regression coefficients with shrinkage factor of 0.77, the c-statistic of the men-specific model was 0.57 (95% CI 0.53–0.60). The 5-year and 10-year calibration plots showed good correspondence between predicted and observed risk (Supplemental Figure 5). The mean predicted 5-year absolute risk of ASAH was 0.04% and ranged from the minimum of 0.02% to the maximum of 0.08%. The mean predicted 10-year cumulative absolute risk was 0.10% and ranged from 0.06% to 0.18%.

### Performance of the original SMA^2^SH^2^ERS risk prediction model in women and men separately

The c-statistic of the original SMA^2^SH^2^ERS risk prediction model in women was 0.63 (95% CI 0.60–0.65). The c-statistic did not differ from the c-statistic of the women-specific model (ΔC –0.00, 95% CI -0.04–0.04). The 5-year and 10-year calibration plots of the SMA^2^SH^2^ERS risk prediction model showed good correspondence between predicted and observed risks (Supplemental Figure 6). The mean predicted 5-year absolute risk of ASAH was 0.06% (range from 0.02% to 0.31%), and the mean predicted 10-year cumulative absolute risk was 0.14% (range from 0.03% to 0.72%) in women.

The c-statistic of the original SMA^2^SH^2^ERS risk prediction model in men was 0.59 (95% CI 0.56–0.63). The c-statistic did not differ of the c-statistic of the men-specific model (ΔC -0.02, 95% CI -0.07–0.02). The 5-year and 10-year calibration plots of the SMA^2^SH^2^ERS risk prediction model showed good correspondence between predicted and observed risks (Supplemental Figure 7). The mean predicted 5-year absolute risk of ASAH 0.02% (range from 0.01% to 0.23%) in men. The mean predicted 10-year cumulative absolute risk was 0.04% (range from 0.03% to 0.08%) in men.

The performance of the original SMA^2^SH^2^ERS risk prediction model was not different between women and men (ΔC 0.02, 95% CI -0.02–0.06).

## Discussion

We developed prediction models, separately for women and men, for estimating absolute risk of ASAH. The sex-specific models performed similarly to the original, non-sex-specific SMA^2^SH^2^ERS risk prediction model,^8^ but had fewer predictors. In women, the c-statistic of the women-specific model was 0.63 and the c-statistic of the original SMA^2^SH^2^ERS risk prediction model was 0.63. In men, the c-statistic of the men-specific model was 0.57 and the c-statistic of the original SMA^2^SH^2^ERS risk prediction model was 0.59. The c-statistic values for all models were low, indicating limited discrimination in both sexes. The original SMA^2^SH^2^ERS risk prediction model showed similar performance in women and in men.

We were not able to find any other sex-specific prediction models of ASAH in the general population to compare our results with. Notably, the original SMA^2^SH^2^ERS risk prediction model contains sex interactions of age, hypertension, and smoking, suggesting different effect sizes of these predictors depending on sex. Here, we found that different variables predicted ASAH in women and men. Age and smoking status were independent predictors of ASAH in both sexes, whereas family history of stroke, alcohol consumption, and educational attainment were independent predictors solely in women, while hypertension was an independent predictor solely in men.

Despite not being an etiological study, our findings are comparable to previous studies on sex differences in the effects of age, hypertension, and smoking. Age is a known risk factor for ASAH in both sexes,^12^ however, increasing age seems to be a risk factor for women only after age of 50 years, after which the sex difference in ASAH incidence emerges.^13^ It has been hypothesized that hormonal and other changes during and after menopause in women aged 50 and above increase the risk of UIA formation and rupture,^7,14^ leading to higher incidence in women. In line with our findings, smoking is in the previous literature an established ASAH risk factor for both sexes, albeit its effects are higher in women.^5^ Interestingly, previous literature shows hypertension to be a similar risk factor in both sexes,^4^ which is contradicting to our findings.

According to our findings, some ASAH predictors seem to be specific to female sex. We found family history of stroke, educational attainment, and alcohol consumption to be independent predictors in women, but not in men. Previous studies have found that family history of ASAH is associated with increased risk of ASAH especially in women,^12^ which is comparable to our findings. Noteworthy, as we did not have information on family history of ASAH, we used family history of any type of stroke as a proxy. In previous literature, low socioeconomic status, which we measured as educational attainment, is known to associate with increased ASAH risk,^15^ but it is unknown if the association differs between sexes. In contrast to our findings, previous studies suggest alcohol consumption to be a risk factor for ASAH in men, but not in women.^4^ However, in our women-specific prediction model, never using alcohol seemed to be stronger predictor for ASAH than using it daily, when occasional use was the reference group. This finding might suggest a protective effect of moderate alcohol use in women. Naturally, etiological studies are needed to investigate this further.

Our models were based on a set of predictors that are routinely available or easily ascertainable in a primary health care setting. We did not include other sex hormone -related predictors than HRT as it was chosen in the development of the original SMA^2^SH^2^ERS risk prediction model, and we wanted to test the same predictors. We did not find HRT to be an ASAH predictor in women. Similarly, previous research has not associated HRT use with increased ASAH risk.^7^ Furthermore, previous studies are contradicting about other hormone-related women-specific risk factors,^7,12,14^ which supports our decision not to include several hormone-related predictors. No genetic predictors were included in our models, as these are not routinely available in health care settings. Previous literature suggests that the genetic risks for ASAH might be different between women and men,^4^ which indicates that inclusion of them in sex-specific prediction models might be worth studying, if they only were available.

Sex itself is a strong risk factor for ASAH. When models are developed separately for women and men the effect of sex is not included as a predictor in the models. Furthermore, there are several sex*predictor interactions in our original SMA^2^SH^2^ERS risk prediction model suggesting sex to be an effect modifier for these predictors. While the sex-specific models perform similarly to the non-sex-specific SMA^2^SH^2^ERS risk prediction model, offer fewer predictors, and show different predictors and prediction sizes for women and men, the original SMA^2^SH^2^ERS model considers the multiplicative interaction between female sex and age, hypertension, and smoking.

An important strength of our study is the large prospective UKBB cohort study with follow-up data on the outcome based on ICD codes from which we derived our sex-specific models. The large sample size enabled us to develop models separately for women and men, despite ASAH being a rare disease. However, participants in the UKBB are more likely to be women, to be of older age and have higher socioeconomical status compared with non-participants.^16^ In addition, the tested predictors are routinely available or easily ascertainable by general practitioners during a standard consultation.

Limitations are, first, that we did not conduct external validation of the sex-specific models as we could not find a validation cohort big enough. The number of ASAH cases was already low, especially among men, in the development cohort and for external validation we would have needed a population-based prospective cohort including the same predictors and as long follow-up at least the size of the UKBB study. Second, some data were missing, and we excluded these participants from the analysis similarly to the original SMA^2^SH^2^ERS study. However, the percentage of missing data in our study was small and likely missing at random, therefore, we expected its potential impact to be negligible. Third, not much is known on the accuracy of identifying incident ASAH in the population cohorts, such as the UKBB. A precious study assessing a limited number of ASAH cases showed the positive predictive value of an ICD code for ASAH in the UKBB to be around 70%,^17^ but further studies are needed. Moreover, it is uncertain if the ICD codes used in the population cohorts solely include ASAH or also non-aneurysmal cases, for example ICD-10 I608 and I609.^18^ We included these codes because their number accounted for over 50% of the cases in UKBB, making it unlikely that these cases were solely non-aneurysmal, as these codes should compromise 10-15% of subarachnoid hemorrhages.^1^ In the original SMA^2^SH^2^ERS study, we performed a sensitivity analysis in the UK Biobank without ICD-10 I608 and I609 and found that the model had a slightly higher c-statistic, but a lower statistical power as it was based on fewer cases. Fourth, the relatively low incidence of ASAH causes outcome imbalance, which we did not correct for based on contradicting findings in previous literature.^19^ Furthermore, the low number of ASAH cases contrasts with the relatively prevalent predictors for this disease. Last, we did not develop ethnicity-specific models as there are not many different ethnicities in UKBB population. A previous study demonstrated that prediction model performance might be different not only between women and men, but also between ethnicities.^9^ Previous literature has found differences in ASAH risk between ethnicities.^20^

Overall, the sex-specific models performed similarly to the general SMA^2^SH^2^ERS risk prediction model in identifying persons at high risk for ASAH. The sex-specific models included fewer predictors than the original prediction model, which could make them easier to use. However, their performance remained suboptimal and did not demonstrate strong discriminatory ability. Like the original SMA^2^SH^2^ERS risk prediction model, the sex-specific models, in their current form, are not suitable for clinical practice. Further validation of the models is required. Furthermore, more research is needed to assess the performance of ethnicity-specific prediction models for ASAH, ideally incorporating an inter-sectional approach that accounts for sex differences.

## Supplemental Material

sj-docx-1-wso-10.1177_17474930251349928 – Supplemental material for Sex-specific risk prediction models for aneurysmal subarachnoid hemorrhage—A UK Biobank study Supplemental material, sj-docx-1-wso-10.1177_17474930251349928 for Sex-specific risk prediction models for aneurysmal subarachnoid hemorrhage—A UK Biobank study by Ina Rissanen, Vita M Klieverik, Jos P Kanning, Mirjam I Geerlings and Ynte M Ruigrok in International Journal of Stroke
