# Supplementary material for: Sex-specific risk prediction models for aneurysmal subarachnoid hemorrhage—A UK Biobank study
Source: Int J Stroke. 2025 Jun 7;20(10):1255–62. doi: 10.1177/17474930251349928 (PMC12664918; doi:10.1177/17474930251349928)
Supplement: sj-docx-1-wso-10.1177_17474930251349928 – Supplemental material for Sex-specific risk prediction models for aneurysmal subarachnoid hemorrhage—A UK Biobank study [file sj-docx-1-wso-10.1177_17474930251349928.docx]

**SUPPLEMENTAL MATERIAL**

**Supplement Table 1** Missing data on sex, predictor variables, and outcome variables.

| Variable | Missing n (%) |
| --- | --- |
| Sex | 1 (0.0%) |
| Age | 1 (0.0%) |
| Family history of stroke | 0 (0.0%) |
| Hypertension | 2031 (%) |
| Smoking status | 2992 (0.6%) |
| Hypercholesterolemia | 3808 (%) |
| Regular physical activity | 27 596 (5.5%) |
| Hormone replacement therapy | 1626 (0.3%) |
| Diabetes mellitus | 923 (0.2%) |
| Alcohol consumption | 1489 (0.3%) |
| Educational attainment | 9041 (2.0%) |
| ASAH outcome | 0 (0.0%) |
| Duration of follow-up | 1 (0.0%) |

ASAH= aneurysmal subarachnoid haemorrhage.

**Supplement Figure 1** Flow chart of the sample selection.

**Supplement Figure 2** Scaled Schoenfeld residuals plots in women.

A) Survival time on linear time scale:

B) Survival time on Kaplan-Meier-transformed time scale:

C) Survival time on logarithmic-transformed time scale:

**Supplement Figure 3** Scaled Schoenfeld residuals plots in men.

A) Survival time on linear time scale:

B) Survival time on Kaplan-Meier-transformed time scale:

C) Survival time on logarithmic-transformed time scale:

**Supplement Figure 4** Calibration plots of predicted and observed probabilities of the women-specific model in women at

A) five years


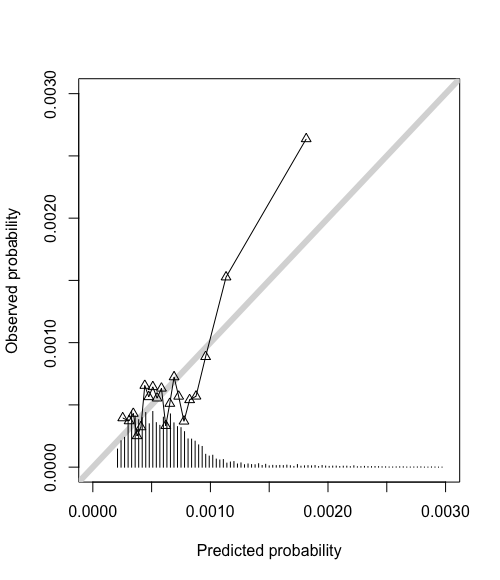


B) ten years

**
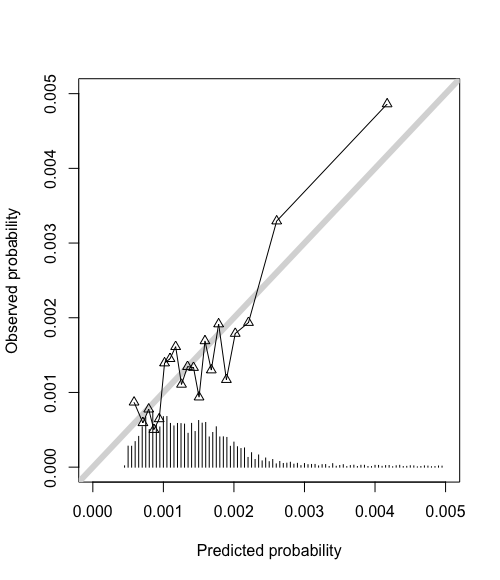
**

**Supplement Figure 5** Calibration plots of predicted and observed probabilities of the men-specific model in men at

A) five years


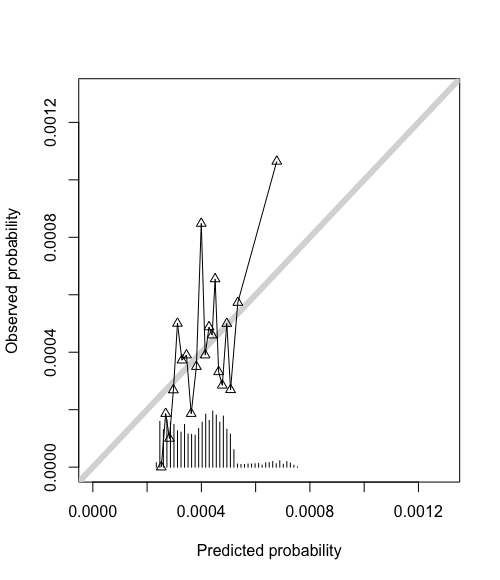


B) ten years


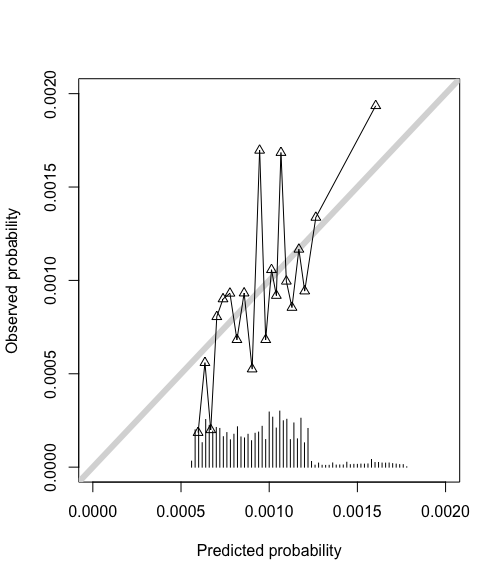


**Supplement Figure 6** Calibration plots of predicted and observed probabilities of the SMA^2^SH^2^ERS calculator in women at

A) five years

**
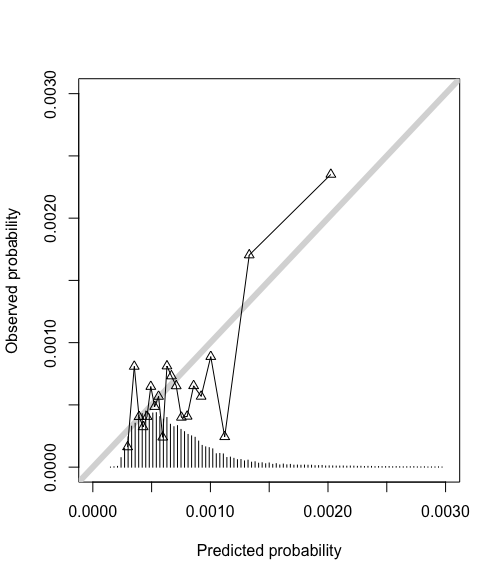
**

B) ten years

**
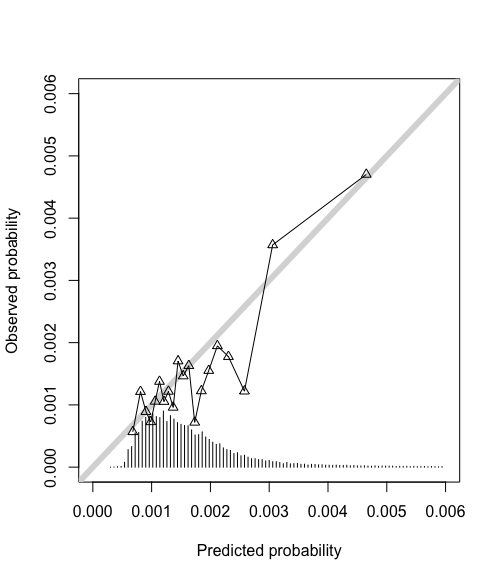
**

**Supplement Figure 7** Calibration plots of predicted and observed probabilities of the SMA^2^SH^2^ERS calculator among men at

A) five years


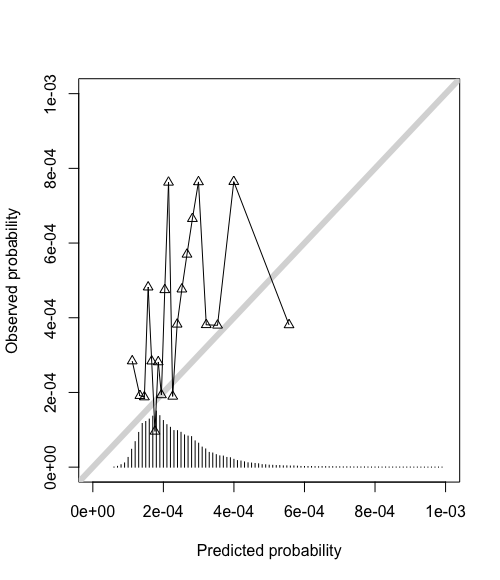


B) ten years

**
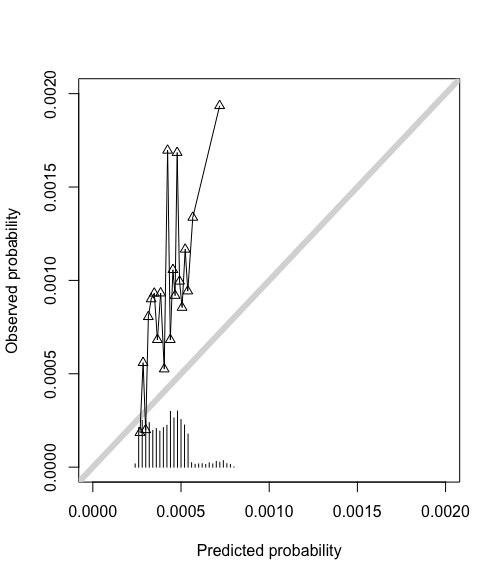
**
